# The Nationalisation of the Children

**Published:** 1901-01-19

**Authors:** 


### THE NATIONALISATION OF THE CHILDREN.

WE urged last week that a nation may with advantage take some care of its children's teeth even though the individualists may be thereby led to raise the cry of "socialism." Mr. Jonathan Hutchinson[1] goes much further and boldly advocates on medical grounds what he calls the nationalisation of the children, and there is a good deal of truth in what he says. He insists "with all the vehemence which is possible upon the physical aspects of the question—that the strength of a nation consists not in its wealth, nor in the extent of its dominion, but in its population. If the population be scanty and made up of weakly units, that nation must become weak and its decline is sure." As medical men we know, he says, what interference with the course of nature in matters pertaining to sex involves. We know that to forbid marriage is simply to encourage immorality; that it is the fruitful parent of various forms of disease; and that the adoption by married people of artifical means to prevent the production of offspring is in the long run prejudicial alike to physical and moral health; while as students of the laws of inheritance we are assured that such customs must injure the prospects of any race that adopts them. "Nothing can be clearer than that if the more prudent part of the population declines to take its share in the production of the next generation it will gradually be supplanted by the more careless. A deterioration in quality will certainly result." Underlying this argument, of course, is the assumption that the prudent and the careful people who by dint of economising, even in the production of children, succeed in the world, are the salt of the earth. This, however, is quite open to question. Between man and man, in a community where law prevails and where the acquisitions even of the feeble are protected, no doubt far-seeing prudence will prevail. But between nation and nation, and, in cir cumstances in which possessions can only be held by force, the matter is rather different, and it is by no means clear that a nation will not stand better among other nations by breeding from reckless dare devils, however low in the social scale, than by encouraging "the more prudent part of the population" to people the earth and possess it. Mr. Hutchinson would begin with free meals for all school children whatever the position of the parents, but he would not end there. The children should be the care of the State, and the more children we have the better. "In every department of English life labour is dear, and if there were more of it it would be better for all." No attempt then should be made to control the production of children. Prolificacy used to be regarded as a blessing, and there is nothing in our modern civilisation, except the love of luxury, which should lead to its being otherwise accounted now. "It is time that this civilisation of ours should face its facts, and, instead of wringing its hands over the improvident marriages of the poor, should recognise that what is wanted is not repression of child-production, but equalisation of the burden of child-rearing." Mr. Hutchinson's opinions deserve every consideration, although we must confess that he hardly seems to give due consideration to the fact that "this civilisation of ours" besides interfering with child-production has also done much to interfere with Nature's processes for the elimination of the unfit. If we go out of our way to encourage the production of all sorts and conditions of children, and at the same time block the action of those natural laws in accordance with which the lame, the halt, the blind, and, be it added, the toothless, would quickly die but for our intervention, it is by no means certain that the race would improve under such fostering care. Mr. Hutchinson appeals to the Spartans. But the Spartans are credited with doing other things to their children besides feeding them at a common table.

[1] Polyclinic, January 1901.

